# Pannexin1 Mediates Early-Life Seizure-Induced Social Behavior Deficits

**DOI:** 10.1080/17590914.2024.2371164

**Published:** 2024-07-16

**Authors:** Price Obot, Antonio Cibelli, Jian Pan, Libor Velíšek, Jana Velíšková, Eliana Scemes

**Affiliations:** a Department of Cell Biology and Anatomy, New York Medical College, Valhalla, New York, USA; b Department of Neuroscience, Albert Einstein College of Medicine, Bronx, New York, USA; c Department of Neurology, New York Medical College, Valhalla, New York, USA; d Department of Pediatrics, New York Medical College, Valhalla, New York, USA; e Department of Obstetrics and Gynecology, New York Medical College, Valhalla, New York, USA

**Keywords:** astrocytes, ion channel, purinergic signaling, sociability, spatial memory, status epilepticus

## Abstract

There is a high co-morbidity between childhood epilepsy and autism spectrum disorder (ASD), with age of seizure onset being a critical determinant of behavioral outcomes. The interplay between these comorbidities has been investigated in animal models with results showing that the induction of seizures at early post-natal ages leads to learning and memory deficits and to autistic-like behavior in adulthood. Modifications of the excitation/inhibition (glutamate/GABA, ATP/adenosine) balance that follows early-life seizures (ELS) are thought to be the physiological events that underlie neuropsychiatric and neurodevelopmental disorders. Although alterations in purinergic/adenosinergic signaling have been implicated in seizures and ASD, it is unknown whether the ATP release channels, Pannexin1 (Panx1), contribute to ELS-induced behavior changes. To tackle this question, we used the ELS-kainic acid model in transgenic mice with global and cell type specific deletion of Panx1 to evaluate whether these channels were involved in behavioral deficits that occur later in life. Our studies show that ELS results in Panx1 dependent social behavior deficits and also in poor performance in a spatial memory test that does not involve Panx1. These findings provide support for a link between ELS and adult behavioral deficits. Moreover, we identify neuronal and not astrocyte Panx1 as a potential target to specifically limit astrogliosis and social behavioral deficits resultant from early-life seizures.

## Introduction

Seizures during childhood are commonly associated with various co-morbidities including cognitive and learning disabilities, anxiety disorders, hyperactivity, and social behavior impairments. Such deficits are also observed in rodents experiencing prolonged seizures during developmental period (reviewed in Holmes, 2016). Interestingly, animal studies indicate that the consequences of prolonged, severe seizures such as status epilepticus (SE) are quite different in the developing and mature brains. While in adult animals SE causes hippocampal extensive neuronal loss with mossy fibers sprouting, young immature animals are more resistant to hippocampal damage after experiencing seizures of similar duration and severity (Haas et al., 2001; Stafstrom, 2002; Velíšková et al., 2018). However, even in the absence of seizure-induced structural damage, important physiological changes occur in the immature brain leading to behavioral changes that can persist into adulthood, including altered level of anxiety, deficits in social behavior and in learning and memory (Bernard et al., 2015; Karnam et al., 2009; Lugo et al., 2014; Rutten et al., 2002; Sayin et al., 2004; Smith et al., 2017), deficits that often fall into autism spectrum disorder (ASD) and intellectual disability (ID) symptomology. Many of the molecular alterations associated with early-life seizures (ELS) such as changes in the expression levels of GABA and glutamate receptors and transporters, ion channels, and kinases (Bernard et al., 2013; Cornejo et al., 2007; Swann, Le, & Lee, 2007; Swann, Le, Lam, et al., 2007), modify the excitation/inhibition (E/I) balance which is thought to underlie neuropsychiatric and neurodevelopmental disorders (Selten et al., 2018; Velíšková et al., 2018).

Besides glutamate and GABA, other neurotransmitters/modulators have been found to also contribute to E/I balance. It is now well established that, in seizure pathology, neuronal excitability can be determined by the ATP/adenosine balance with ATP having an excitatory role (Engel et al., 2012) and adenosine being a negative modulator of hyperexcitability (Dragunow & Goddard, 1984; Dunwiddie & Hoffer, 1980). Release of purines into the extracellular space occurs during and following acute CNS insults (Dale & Frenguelli, 2009), and the level of extracellular ATP, which under physiological conditions is quite low (nanomolar), can reach micromolar levels during pathological conditions. Indeed, increased extracellular levels of ATP following and during seizures have been recorded from brain slices preparations (Doná et al., 2016; Dossi et al., 2018; Santiago et al., 2011). Among the mechanisms by which ATP is released from non-lytic cells, pannexin1 (Panx1) channels have emerged as the main conduit for ATP release from cells (Bao et al., 2004; Dahl, 2015; Locovei et al., 2006; Wang et al., 2014; Wang & Dahl, 2018), also reviewed in Bhat and Sajjad (2021), particularly during seizures (Dossi et al., 2018; Santiago et al., 2011; Vasile et al., 2022). Thus, these channels can be regarded as an important component of the purinergic signaling that contributes to E/I imbalance. Pannexin1 (Panx1) has been shown to form plasma membrane channels that can be activated by different mechanisms including mechanical stretch, membrane depolarization, and following membrane receptor stimulation [reviewed in Lohman et al., (2019)]. In the central nervous system (CNS) Panx1 is expressed in neurons and glial cells (Boassa et al., 2015; Bruzzone et al., 2003; Hanstein et al., 2013); and Panx1 channels have been shown to contribute to neuronal excitation under physiological and pathological conditions (Gajardo et al., 2018; Guzman & Gerevich, 2016; Jourdain et al., 2007; Thompson et al., 2008).

In addition to being a major player in seizure pathology (Cieślak et al., 2017); reviewed in Wong and Engel (2023), purinergic signaling has also been proposed to contribute to pathophysiology of ASD phenotype (Hirsch et al., 2020; Naviaux et al., 2014, 2015, 2017). However, whether purinergic signaling, and in particular Panx1, plays a role in the behavioral deficits that follow ELS is unknown. To examine these issues, we used Panx1 transgenic mice, with global and cell-type specific Panx1 knockout, to evaluate the cognitive and behavioral consequences of ELS using the kainic acid (KA)-induced status epilepticus (SE) model. Our results provide support that ELS leads to deficits in socialization and in the performance of learning and memory tasks. More importantly, they show for the first time that the deficits in social behavior but not deficits in spatial memory are dependent on Panx1 and identify neuronal Panx1 channels as the main contributors of injury induced astrogliosis and social behavior deficits.

## Material and Methods

### Ethical Statement

All mouse studies were done according to National Institutes of Health and Institutional Animal Care and Use Committee guidelines. All procedures were approved by IACUC (approval 54-2-01819H and 71-2-0719) and animal welfare assurance #A3362-01 is on file with the Office of Laboratory Animal Welfare.

### Animals

Panx1 transgenic mice (Panx1^f/f^, global Panx1 KO, GFAP-Cre:Panx1^f/f^ and NFH-Cre:Panx1^f/f^, previously described and characterized: (Hanstein et al., 2013; Hanstein et al., 2016; Obot et al., 2021; Obot et al., 2023; Scemes et al., 2019), were housed in individually ventilated cages containing 3–4 males or females located in a single pathogen-free room in an AAALAC accredited facility. Mice were maintained under 12h light/dark cycle (light on: 7:00 AM, light off: 7:00 PM) and fed standard mouse chow. Food and water were provided to the mice *ad libitum*.

### Early-Life Seizures

#### Kainic acid-Induced Seizures

Post-natal day 21 (P21) mice were injected intraperitoneally (i.p.) with kainic acid (KA; 20 mg/kg; Tocris) and maintained in a controlled environment for observation of seizures, as described (Obot et al., 2021; Scemes et al., 2019). Because mice lacking astrocyte Panx1 have faster onset of forelimb clonus and worse seizure scores than mice with global or neuronal specific deletion of Panx1 (Obot et al., 2021; Scemes et al., 2019), we reduced the dose of KA injected into GFAP-Cre:Panx1^f/f^ mice from 20 to 18 mg/kg. Seizure monitoring was done for up to 120 min after KA injection, when seizures subsided. Two independent observers evaluated KA-induced seizure behavior in mice using a scoring system previously described (Santiago et al., 2011; Scemes et al., 2019). Control mice received saline instead of KA. The selected KA doses produce sustainable status epilepticus (SE; defined as continuous forelimb clonus for >30 min) and had the lowest mortality during the observation period. Given that the duration and magnitude of seizures are related to the extent of behavioral deficits (Austin et al., 2002; Kariuki et al., 2016), only mice that displayed SE were included in the experimental group. After seizures subsided, mice were returned to their home cages and their health monitored daily for 1–2 months, until behavioral testing. The control (saline injected) groups consisted of 45 Panx1^f/f^, 36 global Panx1 KO, 27 NFH-Cre:Panx1^f/f^, and 35 GFAP-Cre:Panx1^f/f^ mice; the SE (KA-injected) groups consisted of 37 Panx1^f/f^, 29 global Panx1 KO, 15 NFH-Cre:Panx1^f/f^, and 14 GFAP-Cre:Panx1^f/f^ mice. As detailed below and in Results, distinct cohorts of control and SE mice were used for behavioral studies. After experimentation, all mice were euthanized and brains from a subset of 2 month old mice harvested for protein analysis.

#### EEG Recordings

Evaluation of whether KA-induced SE resulted in epilepsy later in life, 4 Panx1^f/f^ and 4 global Panx1 KO mice of both sexes that displayed SE for more than 40 min at P21 were implanted at P47 with electroencephalography (EEG) electrodes using stereotactic apparatus (Heinrich Kopf, Inc.), as described (Obot et al., 2021). Continuous EEG/video monitoring was performed for two consecutive weeks using Sirenia system (Pinnacle Technology) to determine the presence of any spontaneous ictal activity. Mice were euthanized painlessly after EEG/video monitoring.

### Behavioral Studies

For behavioral experiments, 2 months old male and female mice were used. The experiments were performed during the light cycle at a time particular to each test, as described below. Animals had free access to water and chow, except for 2 days prior to the radial maze testing when mice received a restricted diet (see below). Behavioral studies were performed by 3 investigators that were blind to the condition (control/SE) and genotype of the mice. As two-ways ANOVA showed that neither the sex nor interactions of sex with the experimental groups had any statistically significant effect (Supplemental Figures 1S–3S), data from male and female mice were combined for behavioral analysis.

#### Open Field

We used the open field to determine the general locomotor activity levels (velocity, total distance travelled, ambulatory episodes), thigmotaxis (tendency to remain close to the walls as a proxy of anxiety-like behavior), and repetitive-like behavior (stereotypic counts). Behavioral testing was performed during 9:00 AM–12:00 PM. Animals (control and SE groups) were placed for 10 min in an arena (43.5 cm × 43.5 cm) with infra-red sensors attached to a computer. Mouse activity in the open field was recorded using Activities^®^ software.

#### Marble Burying

This test evaluates repetitive, restricted behaviors. Alterations on the rodent normal burying behavior is indicative of a perseverative behavior (Malkova et al., 2012; McFarlane et al., 2008). Ten min after open field test, control and ELS mice were placed individually for 15 min in a clean house cage containing 10 marbles placed on the top of the bedding (6 inches high) equidistant from each other in a 3 × 4 arrangement. After removing the mice from the cage, the number of buried marbles was counted. Marbles were considered buried if >50% of the marble was covered by the bedding.

#### Social Interaction

We used the three-chamber test, which measures the motivation of a rodent to interact and engage with its peers (Kaidanovich-Beilin et al., 2011), to assess whether ELS caused social interaction deficits (a core symptom of ASD). Twenty-four hours after being tested in the open field, a subset of control and ELS mice were assessed for social interaction. The three-chamber apparatus consisted in a Plexiglas arena (76.2 cm × 45.5 cm) divided in 3 equal compartments (25.4 cm × 45.5 cm) interconnected by opening (5.0 cm × 6.0 cm) to allow the mice to move freely between the compartments. Metal mesh enclosures were placed into the side compartments and a sniffing zone assigned around them using the AnyMaze software. Testing consisted of two phases: habituation and sociability. For the habituation phase, the test mouse (subject) was placed in the center compartment and allowed to explore for 10 min the whole arena (with empty enclosures placed in each of the two side compartments). During the sociability phase, the subject was allowed to explore the three compartments of the apparatus now containing one empty enclosure (referred to as object) and one enclosure with an unfamiliar conspecific (referred to as animal or stranger) placed in the two-side compartment; the subject was allowed to freely explore the apparatus for 10 min. An overhead camera connected to a computer equipped with AnyMaze software was used to record the time the subject spent in each of the two sniffing zones surrounding the enclosures. Social preference measured according to the method of Yang et al., (2011) was calculated by measuring the time the subject spent with the conspecific *vs* the time spent with the empty cage (object) and the sociability index calculated as the percent time spent with conspecific divided by the total time spent with the object and conspecific. Lack of preference toward a conspecific suggests diminished sociability.

#### Eight Arm Radial Maze

For this experiment, which occurred during 1:00–5:00 PM, a different set of 2 months old mice (not subjected to open field arena or to the marble burying test) was established consisting of control (saline injected at day P21) groups of 15 Panx1^f/f^ and 21 global Panx1 KO mice, and SE (KA injected at day P21) groups of 16 Panx1^f/f^ and 19 global Panx1 KO mice. To investigate spatial orientation and memory performance in mice, we used an automated eight arm radial maze (Ugo Basile) with central circular area (16 cm) and eight arms (36 cm × 5 cm × 15 cm) that could be accessed by automated sliding doors and operated by AnyMaze video tracking software interface, as described (Obot et al., 2023). Briefly, 2 days before the start of the experiment, mice were put on a restricted diet and their weight maintained at 80–85% of their original weight throughout the experiment. The behavioral test consisted of three sessions: (a) day one habituation session followed by (b) 13 daily training sessions, followed by (c) a single test session 24 hours later. The number of working and reference memory errors (see below) and the number of correct choices (number of first entries into baited arms) were recorded during the training and test sessions. Reference memory errors (RME) consisted in the first entry into a non-baited arm, while working memory errors (WME) consisted of repeated entries into previously visited arms. WME was further divided into correct (WM-C: re-entries into baited arms that have been previously visited) and incorrect (WM-I: re-entries into previously visited un-baited arms) errors, as described by others (Jarrard et al., 1984; Pirchl et al., 2010; Yan et al., 2004). For analysis, the number of WM-C, WM-I, RME and of correct choices obtained during the training and test sessions were normalized to their respective mean values obtained at the first training day (day 1).

### Western Blots

A subset of 2-month-old male and female control and SE mice subjected to the three-chamber test were euthanized and brains harvested for Western blot analyses. Whole brains were sonicated in lysis buffer containing protease inhibitors. Each sample (10 µg protein) was electrophoresed on 4% - 20% gradient sodium dodecyl sulfate-polyacrylamide (SDS) mini-gels (BioRad) and transferred to nitrocellulose membranes. Membranes were blocked with 5% milk and incubated for 70 min with antibodies to rabbit polyclonal anti-GFAP (glia fibrillary acidic protein; 1:5,000; Sigma Cat# SAB4501162, RRID:AB_10746077), rabbit polyclonal anti-Cx43 (connexin43; 1:5,000; Sigma Cat#C6219; RRID: AB_476857), rabbit polyclonal anti-ADK (adenosine kinase; 1 : 5,000; Bethyl Cat# A304-280A, RRID:AB_2620476), rabbit polyclonal anti-P2X7R (purinergic receptor P2X7; 1:500; Alomone Labs Cat #: APR-004, RRID:AB_2040068), and to rabbit monoclonal anti-beta-tubulin HRP conjugated (1:5,000; Abcam cat# 185057). Horse-radish-peroxidase (HRP)-conjugated goat anti-rabbit secondary antibodies were used at 1:5,000 (Santa Cruz Biotechnology Cat# sc-2004, RRID:AB_631746). Membranes were exposed to enhanced chemiluminescent substrate (Millipore) and detected with X-ray films. Densitometric analysis of bands was performed with ImageJ.

### Statistical Analyses

Data were analyzed and graphed using GraphPad Prism (9.5.1). The X^2^ test was used to evaluate the relationship between categorical variables. The Shapiro-Wilk test was used to assess whether non-categorial data came from a normally distributed population prior to further analyses. Non-Gaussian distributed data were analyzed using the non-parametric Kruskal-Wallis’ test followed by Dunn’s multi-comparison test, and the normally distributed data analyzed using parametric test (ANOVA) when homoscedasticity was verified by visual inspection of plots of predicted Y values versus the absolute values of residuals. In the latter case, one-way and two-ways ANOVA with/without repeated measures followed by Šidák’s multiple comparisons test were used for data analyses when appropriate. One-sample *t*-test was used to determine whether the degree of social preference was different from no preference. Statistical significance was considered at *p* < 0.05.

## Results

### Outcomes of KA-Injection in P21 Mice

**Figure 1.**
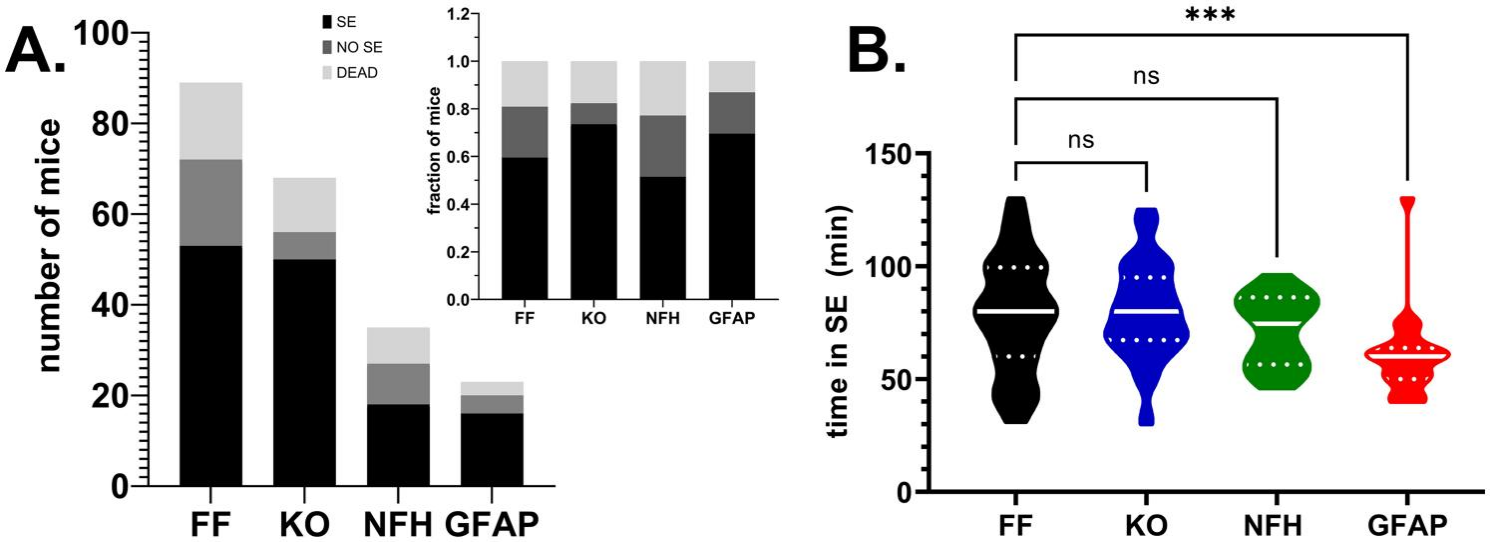
Outcomes of KA injection in P21 mice. **(A)** Number of Panx1^f/f^ (FF), global Panx1 knockout (KO), NFH-cre:Panx1^f/f^ (NFH), and GFAP-cre:Panx1^f/f^ (GFAP) mice that developed status epilepticus (SE; black bars), that did not developed status (NO SE, dark gray bars), and that died (DEAD, light gray bars) after i.p. injection of KA. Inset in **(A)** shows the normalization (fraction of mice) of the outcomes per genotype. **(B)** Violin plot showing the median and quartile values of the time that Panx1^f/f^ (FF; *n* = 53), global Panx1 knockout (KO; *n* = 50), NFH-Cre:Panx1^f/f^ (NFH; *n* = 18), and GFAP-Cre:Panx1^f/f^ (GFAP; *n* = 22) mice spent in SE. ***p = 0.001, ns = not significant (Kruskal-Wallis followed by Dunn’s multiple comparison test).

After i.p. injection of KA, mice of all genotypes developed behavioral seizures; the duration of SE lasted on average 60–80 min. The number of mice that did or did not develop SE, the number of deaths and the mean time spent in SE that occurred after i.p. injection of KA are shown in Figure 1A and B. No significant difference in terms of the outcome (SE, NO SE, DEAD) was detected between the genotypes (X^2^ = 7.97, *p* = 0.24) and no significant difference in the time spent in SE was detected between Panx1^f/f^ and mice with global and neuronal Panx1 deletions (Panx1^f/f^: 78.57 ± 3.76 min, *n* = 53; global Panx1 KO: 79.80 ± 2.99 min, *n* = 50; NFH-Cre:Panx1^f/f^: 71.50 ± 3.69 min, *n* = 18; *p* > 0.05, Dunn’s test). However, as was expected due to the reduced KA dose administered to mice lacking astrocyte Panx1 (see Methods), a significant difference in time spent in SE was detected between Panx1^f/f^ and GFAP-Cre:Panx1^f/f^ mice (Figure 1B; Panx1^f/f^: 78.57 ± 3.76 min, *n* = 53; GFAP-Cre:Panx1^f/f^: 60.36 ± 3.95 min, *n* = 22; Kruskal-Wallis followed by Dunn’s test; *p* = 0.001). Despite the shorter time GFAP-Cre:Panx1^f/f^ mice spent in SE (about 60 min) compared to other genotypes (70 - 80 min), SE mice lacking astrocyte Panx1 also displayed behavior impairments later in life (see below). Analysis of continuous (2 weeks) EEG/video recordings performed 40 days post KA injection in 4 Panx1^f/f^ and 4 global Panx1 KO indicated absence of spontaneous seizures in these mice, which also displayed normal ambulation, appetitive, and exploratory behaviors.

### Panx1 and Early Life Seizure (ELS) Do Not Affect Locomotor Activity and Anxiety-like Behavior

**Figure 2.**
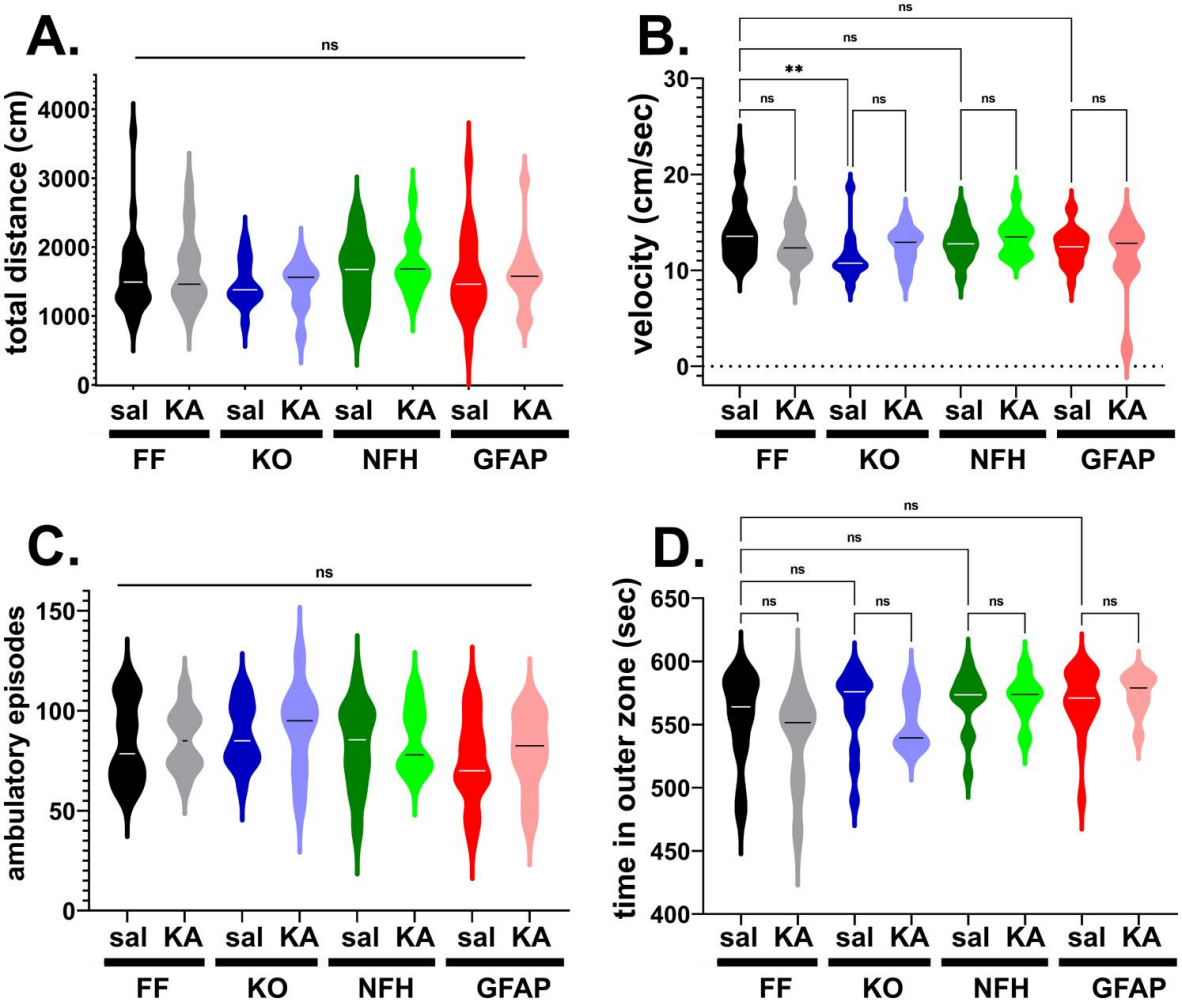
Panx1 and early life seizure do not affect locomotor activity and anxiety-like behavior. Violin plots showing the median values of the **(A)** total distance travelled, **(B)** velocity, **(C)** number of ambulatory episodes, and **(D)** time spent in the outer zone of the open field arena obtained for 2 months old Panx1^f/f^ (FF), global Panx1 knockout (KO), NFH-Cre:Panx1^f/f^ (NFH), and GFAP-Cre:Panx1^f/f^ (GFAP) mice that were injected with saline (Sal) and kainic acid (KA) at age P21. KA groups correspond to mice that developed SE. **p = 0.001, ns = not significant (Kruskal-Wallis followed by Dunn’s test). Number of mice: FF_sal_ (30), FF_KA_ (18), KO_sal_ (15), KO_KA_ (10), NFH_sal_ (14), NFH_KA_ (15), GFAP_sal_ (22), GFAP_KA_ (14).

To evaluate whether Panx1 and/or ELS affected the overall locomotor activity and thigmotaxis (as a proxy for anxiety-like behavior) in adulthood, the open field was used to assess performance of 2 month old mice with global and cell type specific deletion of Panx1 injected with either saline or KA at age P21. Locomotor activity was evaluated by measuring the total distance traveled, velocity, and the number of ambulatory episodes during 10 min in the open field arena. No significant differences in terms of distance travelled and the number of ambulatory episodes were detected between the groups (Figure 2; Kruskal-Wallis; *p* > 0.05); however, a small yet significant difference in the velocity was detected only between saline-injected Panx1^f/f^ and saline-injected global Panx1 KO mice (Figure 2B; *p* = 0.001, Kruskal-Wallis followed by Dunn’s test) but not between genotype-matched ELS and saline-injected mice (Figure 2B; *p* > 0.40 Dunn’s test). With regard to thigmotaxis (time spent in the outer zone), although a significant difference was detected among the groups (*p* = 0.04, Kruskal-Wallis), there were no significant differences detected between the genotypes (saline injected) and between genotype-matched ELS and saline-injected mice (Figure 2D; *p* > 0.19, Dunn’s multi-comparison test).

Given that no overall deficits in locomotor activity and thigmotaxis were recorded in saline- or KA-injected adult mice of all genotypes, our results indicate that neither Panx1 nor ELS lead to anxiety-like behavior.

### Early Life Seizures Lead to Social Behavior Deficits in a Panx1 Dependent Manner

**Figure 3.**
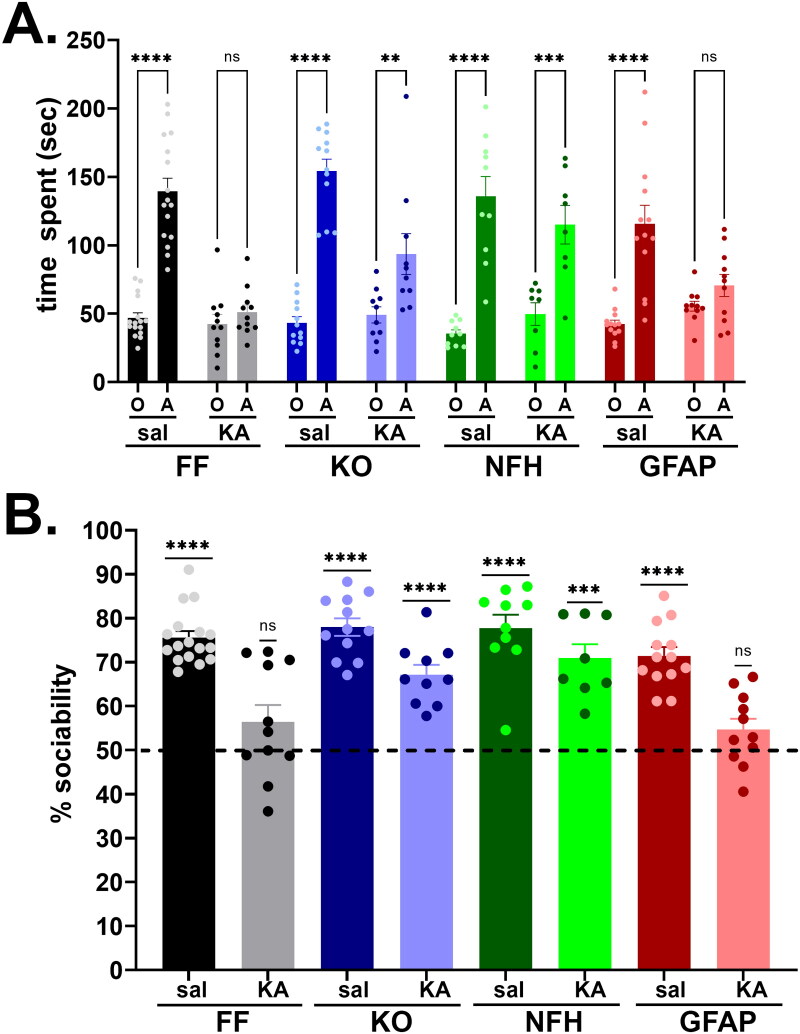
Panx1 dependence of social behavior deficits induced by early life seizures. **(A)** Means ± sem values of the time that 2 months old Panx1^f/f^ (FF), global Panx1 knockout (KO), NFH-Cre:Panx1^f/f^ (NFH), and GFAP-Cre:Panx1^f/f^ (GFAP) mice, injected with saline (Sal) and kainic acid (KA) at age P21, spent with an object (O) and with a conspecific (A) measured during the three-chamber test. ****p < 0.0001, ***p = 0.0001, **p = 0.007, ns = not significant (two ways ANOVA followed by šidák’s multi-comparison test). **(B)** Means ± sem values of the sociability (percent time spent with conspecific to the total time spent with object and conspecific) obtained from 17 FF_sal_, 11 FF_KA_, 12 KO_sal_, 10 KO_KA_, 10 NFH_sal_, 8 NFH_KA_, 13 GFAP_sal_, 11 GFAP_KA_ mice. ****p < 0.0001, ***p = 0.0003 (one sample *t*-test).

To evaluate whether ELS contributes to ASD-like behavior later in life, we assessed mouse sociability using the three-chamber test. Since no sex differences were detected among the groups (Supplemental Figure 1S), male and female mice were combined for analysis of social behavior. Results obtained from 2-month-old mice show that Panx1 plays an important role in the development of social behavior following ELS (Figure 3). Figure 3A shows the mean ± s.e.m. values of the time that control and ELS mice spent with a conspecific and the novel object. Control (saline-injected) mice of all genotypes spent more time (about 75–80%) with a conspecific than with a new object (Figure 3A dark-colored bars), as indicated by two-way ANOVA (F_interaction_ (7, 166) = 9.765, *p* < 0.0001; F_preference_ (1, 166) = 208.0, *p* < 0.0001; F_genotype_ (7, 166) = 7.70, *p* < 0.0001) and post-hoc analysis (*p* < 0.0001, Šidák’s multiple comparison tests). The time spent with the object or with the conspecific did not differ among the genotypes of saline-injected mice (*p* > 0.55, Šidák’s multiple comparison tests). However, following ELS (Figure 3A light-colored bars), the time spent with conspecific decreased in a genotype dependent manner; mice expressing Panx1 (Panx1^f/f^; Figure 3A, gray bars) and mice lacking astrocyte Panx1 (Figure 3A, red bars) had no social preference, i.e., mice spent similar amount of time with conspecific and with object (*p* = 0.99 and *p* = 0.873, Šidák’s multiple comparison test, respectively), while global Panx1 KO mice (Figure 3A, light-purple bars) and mice lacking neuronal Panx1 (Figure 3A, green bars) did not show any social deficit, spending more time with conspecific than with the object (*p* = 0.007 and *p* = 0.0001, Šidák’s multiple comparison test, respectively). Interestingly, although the time spent with the conspecific was reduced in ELS compared to saline-injected global Panx1 KO mice (sal: 154.1 ± 8.76 sec, KA: 93.54 ± 14.91 sec, *p* = 0.0037 Šidák’s multiple comparison test), this decrease was not sufficient to affect their sociability index (Figure 3B), as occurred in Panx1^f/f^ (sal: 139.5 ± 9.56 sec, KA: 51.11 ± 5.28 sec; *p* < 0.0001, Šidák’s multiple comparison test) and GFAP-Gre:Panx1^f/f^ mice (sal: 115.7 ± 13.54, KA: 70.62 ± 8.02 sec; *p* = 0.048, Šidák’s multiple comparison test). Figure 3B shows the sociability index (percent time spent with conspecific relative to the total time spent with both conspecific and object) which indicates that ELS did not affect the sociability index of Panx1 KO and NFH-Cre:Panx1^f/f^ mice (one-sample *t*-tests: *p* < 0.0001 and *p* = 0.0003, respectively) but reduced the degree of sociability of Panx1^f/f^ and GFAP-Cre:Panx1^f/f^ mice to levels not significantly different from what would be expected for no preference (one-sample *t*-tests: *p* = 0.13 and *p* = 0.087, respectively).

Overall, these data show that Panx1 contributes to social behavior impairment following ELS, and that deletion of neuronal but not astrocyte Panx1 confers protection against this ASD-like behavior. Importantly, given that some mice that did not develop SE at P21 albeit KA-injected (as defined in Methods) also did not show ASD-like behaviors later in life (Panx1^f/f^: 74% sociability, *n* = 19; GFAP-Cre:Panx1^f/f^: 82% sociability, *n* = 4; data not shown), it is likely that prolonged (< 30 min) SE is required for Panx1 to exert deleterious effects.

### Repetitive, Stereotypic Behavior

**Figure 4.**
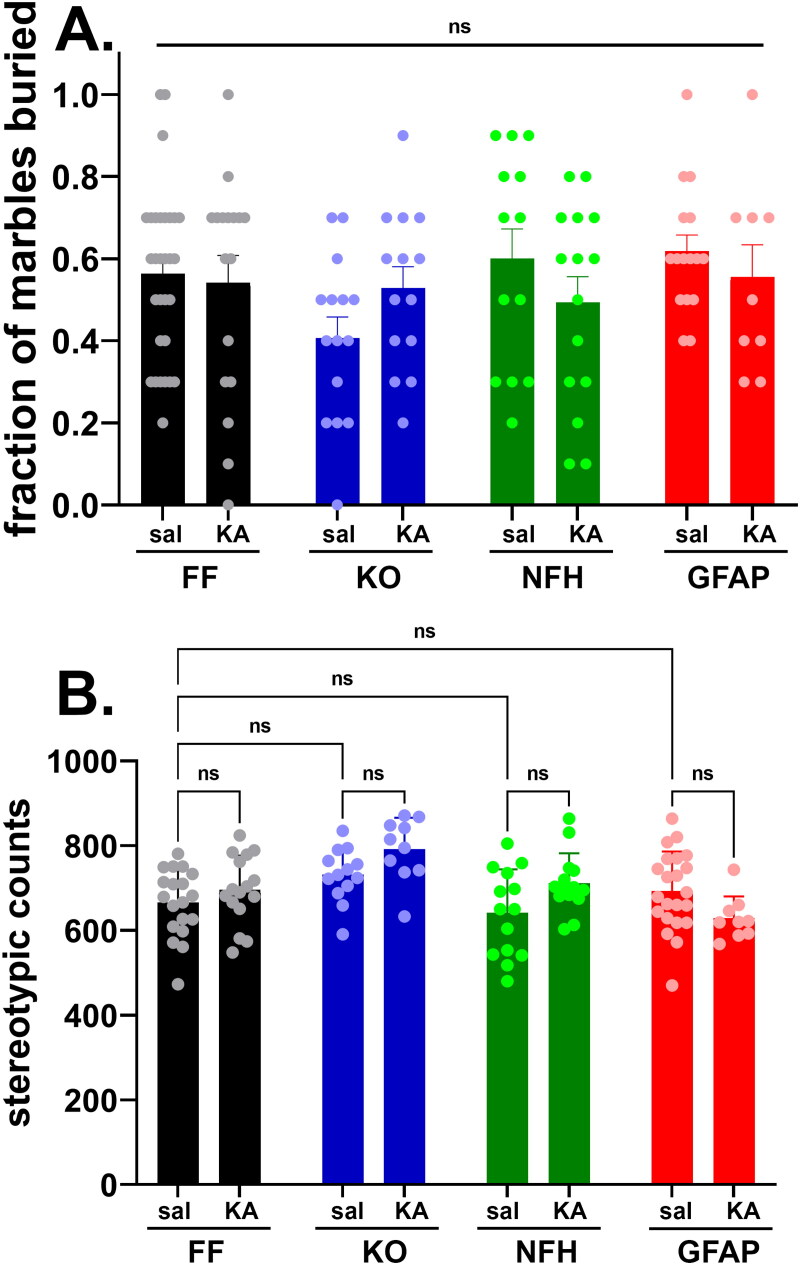
Stereotypic behavior is not altered by Panx1 and early-life seizures. Means ± sem values of the **(A)** fraction of buried marbles and **(B)** number of stereotypic counts measured from 2 months old Panx1^f/f^ (FF), global Panx1 knockout (KO), NFH-Cre:Panx1^f/f^ (NFH), and GFAP-Cre:Panx1^f/f^ (GFAP) mice, injected with saline (Sal) and kainic acid (KA) at age P21. In part **A,** values were obtained from 30 FF_sal_, 17 FF_KA_, 15 KO_sal_, 13 KO_KA_, 10 NFH_sal_, 15 NFH_KA_, 16 GFAP_sal_, 9 GFAP_KA_ mice. In part **B,** values were obtained from 19 FF_sal_, 16FF_KA_, 15 KO_sal_, 14 KO_KA_, 14 NFH_sal_, 14 NFH_KA_, 22 GFAP_sal_, 9 GFAP_KA_ mice. ns = not significant (two ways ANOVA followed by Šidák’s multiple comparison test).

We next evaluated, using the marble burying test, whether Panx1 participates in stereotyped, repetitive behaviors as a result of ELS. Given that there were no sex differences among the groups (Supplemental Figure 2S), results are presented by combining male and female mice. The number of marbles buried (out of a total of 10) by KA- and saline-injected mice were counted, and the fraction of buried marbles used as a measure of repetitive behavior. Both KA- and saline-injected mice of all genotypes maintained similar fractions of buried marbles (Figure 4A; two-way ANOVA: F_interaction_ (3, 121) = 1.408, *p* = 0.2439; F_condition_ (1, 121) = 0.1822, *p* = 0.6703; F_genotype_ (3, 121) = 1.420, *p* = 0.2402), suggesting that neither early CNS insult from SE nor deletion of Panx1 affect burying behavior later in life.

As another measure of repetitive behavior, we evaluated stereotypic counts assessed in the open field. In this case, although a significant difference was detected among the groups (Figure 4B; two-way ANOVA: F_interaction_ = (3, 109) = 3.697, *p* = 0.0140; F_genotype_ = (3, 109) = 7.365, *P* = 0.0002; F_condition_ = (1, 109) = 2.396, *p* = 0.1245), there were no significant differences between the genotypes of control mice (Šidák’s multi-comparison test, *p* > 0.5) and between condition (control and ELS) within each genotype (Šidák’s multi-comparison test, *p* > 0.5). Because the significant differences (Tukey’s multiple comparison tests) observed were between ELS-global Panx1 KO *vs* control-Panx1^f/f^ (*p* = 0.003), *vs* control-GFAP-Cre:Panx1^f/f^ (*p* = 0.0347), *vs* control-NFH-Cre:Panx1^f/f^ (*p* = 0.0005), and *vs* ELS-GFAP-Cre:Panx1^f/f^ (*p* = 0.0007) mice but not between genotypes injected with saline or with KA, we considered that neither Panx1 nor ELS contributed to modify the stereotypic behavior, a finding consistent with that obtained for the marble burying test (see Figure 4A).

### Panx1 Deficiency and Early Life Seizure Lead to Spatial Memory Impairment

Given that global or cell type specific deletion of Panx1 resulted in impaired long-term spatial memory (Obot et al., 2023), we evaluated whether cognitive (learning and memory) deficits associated with ELS (Sayin et al., 2004) were modified by Panx1 expression. For that, we assayed, using the 8-arm radial maze, spatial (working and reference) memory in 2-month-old control and ELS Panx1^f/f^ and global Panx1 KO mice. In addition, we combined male and female mice for analysis of spatial memory, since we found that this cognitive behavior was independent of sex (Supplemental Figure 3S).

**Figure 5.**
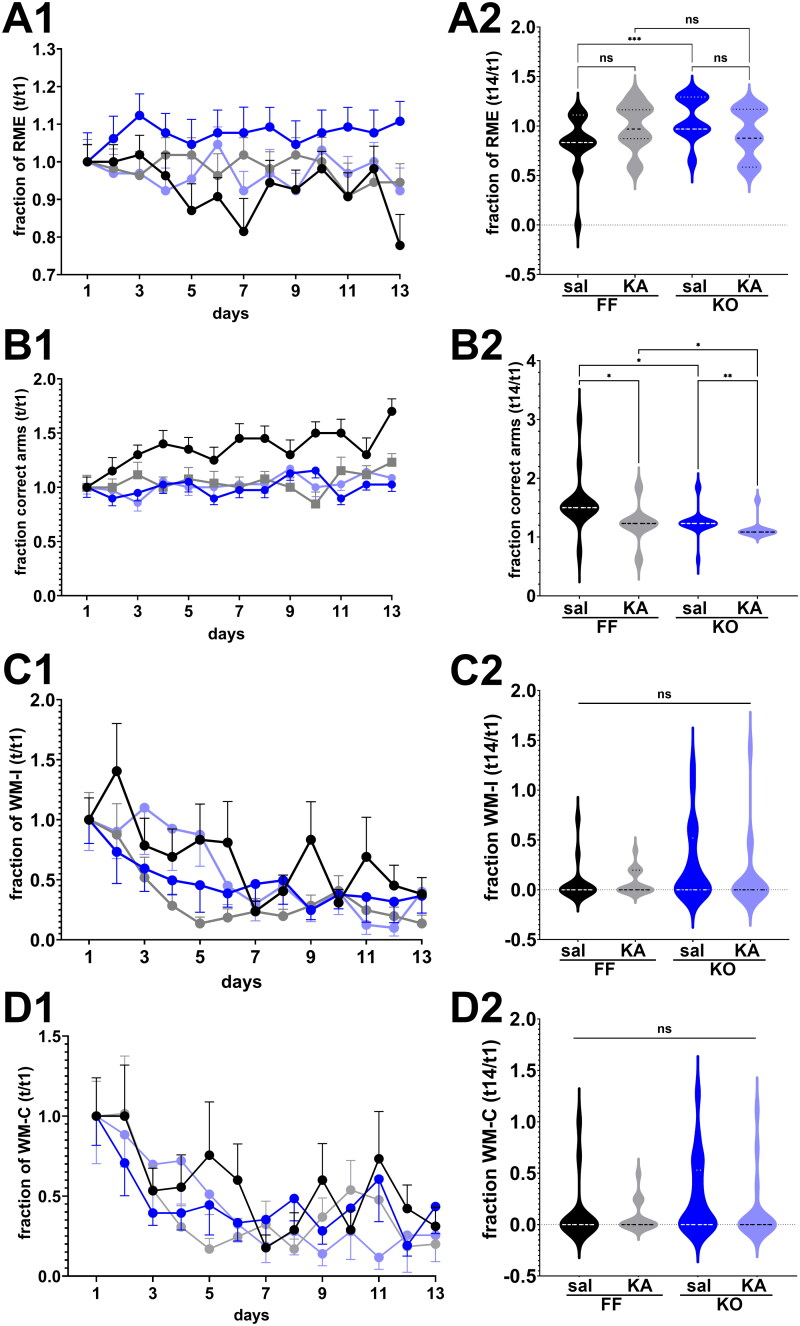
Long-term reference memory impairment. **(A1–D1)** Mean ± sem of the (**A1**) fraction of reference memory errors (RME), (**B1**) correct choices (correct arms), (**C1**) working memory incorrect errors (WM-I), and **(D1**) working memory correct errors (WM-C) obtained during the training sessions. (**A2 - D2**) Violin plot showing the median values of RME (**a**), correct arms (**B**), WM-C (**D**), and WM-I (**C**) obtained during the test phase for 2 months old mice that were saline (Sal) and kainic acid (KA) injected at age P21. Panx1^f/f^ (FF_sal_, *n* = 15; FF_KA_, *n* = 21) and global Panx1 knockout (KO_sal_, *n* = 21; KO_KA_, *n* = 19) mice. ns: not significant, *p < 0.03, **p = 0.007, ***p = 0.0005 (Kruskal-Wallis followed by Dunn’s multiple comparison tests).

Figure 5 shows the fraction of reference and working memory errors and the fraction of correct arms first visited obtained from the four groups of mice recorded during the training (parts A1 – D1) and test sessions (parts A2 – D2). The fraction of reference memory errors (RME) recorded from control and ELS Panx1^f/f^ and global Panx1 KO mice did not vary over time (Figure 5A1; Two-way repeated measure ANOVA: F_time_ (12, 840) = 0.5988, *p* = 0.8442); however, RME differed significantly among the four groups during the 13 days training (Figure 5A1; Two-way repeated measure ANOVA: F_groups_ (3, 67) = 10.44, *p* < 0.0001). At day 14 (test session), pair-wise analysis revealed differences only between saline-injected Panx1^f/f^ and global Panx1 KO mice (Figure 5A2; Kruskal-Wallis, *p* = 0.0027 followed by Dunn’s multiple comparison test: *p* = 0.0011), but not between control (saline-injected) and ELS (KA-injected) Panx1^f/f^ or global Panx1 KO mice (Figure 5A2). In terms of fraction of correct (baited) arms first visited, significant differences were recorded during the 13 days’ training session (Figure 5B1; Two-way repeated measure ANOVA: F_time_ (12, 840) = 2.108, *p* = 0.0145) and among the groups F_groups_ (3, 67) = 34.79, *p* < 0.0001). At day 14, both ELS Panx1^f/f^ and global Panx1 KO mice showed reduced fraction of correct arms visited compared to that obtained from control mice (Figure 5B2; Kruskal-Wallis, *p* < 0.0001; followed by Dunn’s test, *p* < 0.01). These results suggest that ELS has a significant impact on long-term reference memory, at least in terms of correct arms visited.

Unlike long term reference memory, no significant differences were detected in WME among the four groups during the training and test sessions (Figure 5C,D). Figures 5C1, D1 show that the fraction of WM-C and WM-I decreased significantly over time for each mouse genotype (Two-way ANOVA; F_time_ (12, 804) = 7.254, *p* < 0.0001 and F_time_ (12, 840) = 6.626, *p* < 0.0001, respectively) indicating that mice learned the task by reducing the number of errors. However, during the 13 days’ training sessions, there was no significant differences in terms of WM-C and WM-I between the four groups (Two-way repeated measure ANOVA; F_groups_ F (3, 64) = 0.7040, *p* = 0.5532 and F_groups_ F (3, 67) = 2.231, *p* = 0.0926, respectively). At day 14 (test day), WM-C and WM-I did not differ significantly among the groups (Figures 5C2, D2; Kruskal-Wallis, *p* = 0.1095 and *p* = 0.1497, respectively). Thus, these results show that ELS does not have an impact on working memory in adulthood.

Overall, our results indicate that ELS alters long-term reference spatial but not working memory and that Panx1 does not play a role in this process.

### Increased GFAP Expression Levels following ELS is Abrogated in Mice Lacking Neuronal Panx1

**Figure 6.**
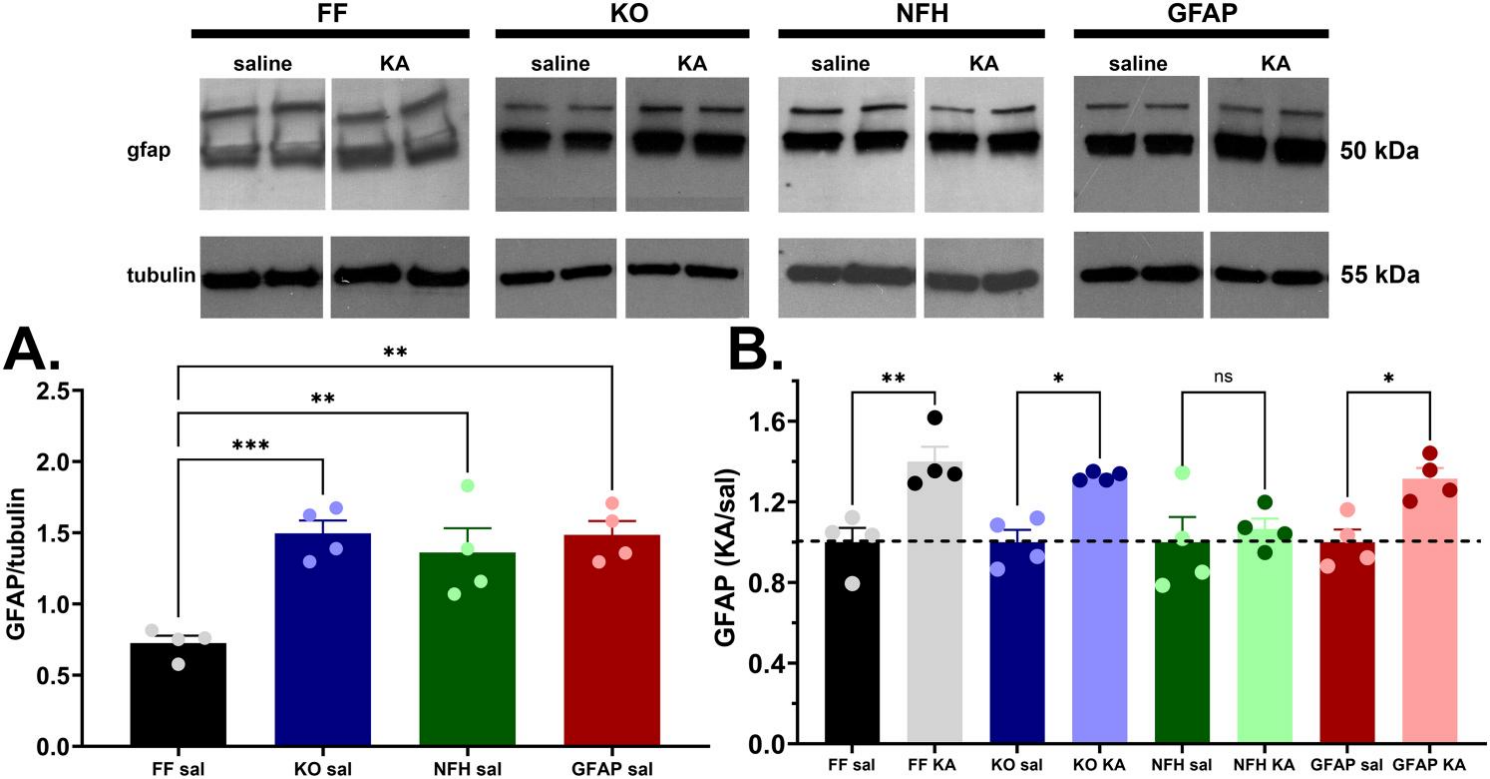
Increased GFAP expression levels following ELS is abrogated in mice lacking neuronal Panx1. (**A**) Mean ± sem of GFAP expression measured from whole brain homogenates of 2-months old control (saline-injected at P21: Sal) Panx1^f/f^ (FF), global Panx1 knockout (KO), GFAP-Cre:Panx1^f/f^ (GFAP), and NFH-Cre:Panx1^f/f^ (NFH) mice. *p = 0.0172, ***p = 0.0007, (one-way ANOVA followed by Dunnett’s multiple comparison tests, *n* = 4 mice per genotype). (**B**) Mean ± sem of the fractional changes in GFAP expression levels measured from whole brains of 2-months old mice that were injected at P21 with kainic acid (KA) relative to saline-injected (Sal) mice. ns = not significant, *p = 0.03, **p = 0.006, (one-way ANOVA followed by Šidák’s multi-comparison test; *n* = 4 mice per group). **Top panel**: Representative Western blots showing GFAP and β-tubulin expression levels in whole brains of control (Sal) and ELS (KA) mice.

Because disruption of normal glia function has been proposed to underlie the occurrence of ASD (reviewed in Gzielo & Nikiforuk, 2021), we evaluated the extent to which ELS led to alterations in the expression levels of some of the main glial markers reported to be altered following seizures (reviewed in Vezzani et al., (2022), that could have contributed to ASD symptoms here reported. For that we quantified the expression levels of adenosine kinase (ADK), purinergic (P2X7) receptor, connexin43 (Cx43), and glia fibrillary acid protein (GFAP) in samples of whole brain homogenates of 2 month old control and ELS-mice. No significant differences in expression levels of ADK, P2X7 receptor and Cx43 were recorded between control and ELS mice of any of the genotypes tested (Suppl. Fig. 4S). In contrast, a significant increase in GFAP expression levels was observed among the genotypes and conditions (Figure 6). Interestingly, GFAP expression in brain lysates of 2 month-old control (saline-injected) Panx1 deficient mice (global and cell-type specific) were about 2.0 fold higher than in Panx1^f/f^ mice (Figure 6A). Despite the increased basal level, GFAP expression in ELS mice was found to be further elevated by about 1.4 fold in all genotypes, except in brains of mice lacking neuronal Panx1 where levels remained similar (1.07-fold) to basal levels (Figure 6B; see also Supplemental Fig. 5S showing that even changing the tubulin X-ray films exposure time, the normalized GFAP levels in NFH-Cre:Panx1^f/f^ samples of KA-injected mice are not statistically different from those of saline injected mice). These results suggest that neuronal Panx1 channels are likely contributors of injury induced astrogliosis that underlies the development of social behavior deficits.

## Discussion

Here we investigated whether Panx1 channels play a role in behavioral impairments following early-life seizures (ELS), using the KA-induced SE in P21 Panx1 deficient mice (global and cell-type specific) and evaluated changes in expression levels of some glia markers that have been shown to be altered following seizures. We found that in adulthood ELS led to deficits of long term-reference memory that was Panx1 independent and to social behavior impairment that was dependent on neuronal Panx1. Neither Panx1 nor ELS influenced thigmotaxis (a proxy for anxiety-like behavior), locomotor activity, and repetitive behaviors. We found that among the glia markers reported to be altered following seizures, only GFAP expression was elevated in all genotypes, except in brains of mice lacking neuronal Panx1. We suggest that the astrogliosis associated with social behavior deficit is mediated by neuronal Panx1 channels.

KA-seizures in immature rodent brains are often not associated with wide-spread neuronal loss, axonal sprouting, and the development of spontaneous recurrent seizures (SRS), as they do if induced in adulthood (Bernard et al., 2015; Cornejo et al., 2007; Stafstrom et al., 1992; 1993; Yang et al., 1998), or if triggered during a critical period identified in rats to be between 3 and 4 weeks of age (Sayin et al., 2015). This is because, differently from other models, KA-seizures require a mature limbic system to induce neuronal damage, which occurs after 3 weeks of age with hippocampal CA3 and dentate hilar neuron loss (Bayer, 1980a, 1980b).

Even without structural damage, ELS has been shown to affect rodent behavior with features similar to autism spectrum disorder (ASD) and intellectual disabilities (ID), common comorbidities of childhood epilepsy. Indeed, the question of whether seizures *per se* can directly influence the development of cognitive and social behavior deficits in adulthood have been addressed in many studies in which rats or mice subjected to KA-, pilocarpine- or flurothyl-induced seizures at postnatal day (P5 – P20) were shown to develop later in life learning and memory deficits, social behavior impairments, and repetitive behaviors (Bernard et al., 2015; Castelhano et al., 2013; Cornejo et al., 2008; Hodges et al., 2019; Karnam et al., 2009; Lugo et al., 2014; Rutten et al., 2002; Sayin et al., 2004; Smith et al., 2017). Although these studies provided some evidence for a causal relationship between ELS and ASD/ID, very few have used more than one behavioral test to examine the three core features of ASD/ID (sociability, communication, repetitive behavior and learning and memory) or examined whether spontaneous recurrent seizures (SRS) influence the phenotype.

Our results obtained from 2 month-old mice, showing that ELS led to spatial learning memory deficits is in agreement with studies showing somewhat similar cognitive impairments, as measured using Morris water maze in adult rats and in mice subjected during young ages (P7-P24) to KA-, pilocarpine-, flurothyl-ELS [reviewed in Stafstrom, 2002; Bernard & Benke, 2015]. Of note, is that the reported deficits in spatial memory of ELS rodents were more prominent during the acquisition but not recall phase of the test (Cornejo et al., 2007), except for one study (Sayin et al., 2004) that found impaired acquisition and recall in KA-ELS rats using the radial and water maze task. Differently from the previous reports, however, the only effect of ELS on spatial memory found here was related to long term reference memory, as measured by the number of correct arms first visited during the test day (recall), but not difference in terms of the number of working and reference memory errors during the training (acquisition) phase. It is unclear whether this discrepancy relates to the animal’s age at ELS induction and assessment, to the ELS protocol, or to differences in spatial memory apparatus used. Nevertheless, our finding showing impaired long term reference memory in both Panx1^f/f^ and global Panx1 KO support the notion that ELS leads to cognitive impairment later in life. Moreover, our data show that the effects of ELS on spatial memory are independent of Panx1 given that global deletion of Panx1 did not ameliorate or worsen memory deficit.

Social interaction and communication deficits, and repetitive - stereotypic behaviors are the main core features of ASD. Here we show using the three-chamber test that ELS led to social behavior impairments in 2 months-old Panx1^f/f^ mice, with mice spending less time with conspecific than non-SE mice. However, with regard to the other core feature of ASD, neither Panx1 nor ELS affected repetitive/stereotypic behaviors, as measured by the number of marbles buried and the stereotypic counts. Our results are similar to those previously obtained in adult rats subjected to a single KA injection to induce SE at P7-P9 (Bernard et al., 2013; Castelhano et al., 2013; Waltereit et al., 2011) and to those in which seizures in mice were induced by flurothyl (Hodges et al., 2019; Lugo et al., 2014). Importantly, our results showing that mice with global or neuronal deletion of Panx1 were protected from ELS-induced social impairment, reveal that the ATP release Panx1 channels play an important role in ELS-induced sociability deficits. The involvement of ATP-purinergic signaling in ASD has been proposed based on the reversal of ASD symptoms in a maternal immune activation mouse model of neurodevelopmental and neuropsychiatric disorders by the broad spectrum P2 receptor blocker, suramin (Naviaux et al., 2014). More recently, it was demonstrated that deficits in ATP release contributed to social behavior and repetitive behavior impairments through its action on P2X2 receptors that modulates GABAergic transmission (Wang et al., 2021).

Previous studies have shown that, in seizure models and in resected tissues of epileptic patients, the ATP release Panx1 channels contribute to seizures by maintaining neuronal hyperexcitability (Aquilino et al., 2020; Dossi et al., 2018; Santiago et al., 2011). In animal models, deletion or blockade of Panx1 ameliorates the behavioral manifestation of seizures (Aquilino et al., 2020; Santiago et al., 2011), reduces the EEG power spectra, particularly in the delta (0.5–5.0 Hz) and beta (13–30 Hz) bands (Obot et al., 2021), frequencies associated with the progression of non-convulsive to convulsive seizures (Sharma et al., 2018). Interestingly, although the selective deletion of Panx1 from neurons or astrocytes resulted in similar attenuation of the EEG power spectra (Obot et al., 2021), they differently affected behavioral seizures: while mice lacking neuronal Panx1 exhibited longer latency to KA-induced SE and less severe behavioral seizures, mice lacking astrocyte Panx1 exhibited shorter latency and more severe seizures (Obot et al., 2021; Scemes et al., 2019). Because the severity and duration of seizures in early life may relate to the extent of behavioral deficits (Stafstrom, 2002), we opted to reduce the KA dose injected into P21 GFAP-Cre:Panx1^f/f^ mice. Here we showed that, by slightly reducing the KA dose, we were able to induce similar seizure outcomes in these mice as in the other three genotypes. Despite the shorter time GFAP-Cre:Panx1^f/f^ mice spent in SE (about 60 min) compared to other genotypes (70 - 80 min), ELS mice lacking astrocyte Panx1 were not protected from social behavior impairments as for mice with global or neuronal Panx1 deletion. These results together with the observation that social behavioral impairments were only present in ELS mice that sustained SE for more than 30 min, show that Panx1 and not the duration of SE determines the extent to which ELS affects social behavior later in life.

A potential mechanism by which ELS induces behavioral deficits in adulthood has been hypothesized to be mediated by glia activation given that in response to acute or chronic CNS insult, astrocytes become prone to gliosis, manifested as increased proliferation and altered morphology with a concomitant upregulation of GFAP staining. Under gliotic conditions, baseline astrocytic functions are disrupted, due to altered expression of crucial proteins (connexins, ion channels, neurotransmitter transporters [reviewed in Nagelhus et al., (2013)]. Of the glial markers tested here (Cx43, ADK, P2X7, and GFAP) that have been reported to have altered expression levels in epileptic tissues, particularly in patients and animal models of temporal lobe epilepsy [reviewed in (Ahn et al., 2023; Aronica et al., 2013; Bedner & Steinhäuser, 2023; Hayatdavoudi et al., 2022)], we found that only the expression level of GFAP was altered in 2 months old ELS mice. The unaltered expression levels of ADK, Cx43, and P2X7 receptors which contribute to the development of epilepsy (Aronica et al., 2011; Henning et al., 2023; Pannasch et al., 2011; Skaper et al., 2010; Song et al., 2019; Wallraff et al., 2006) together with the finding that astrogliosis *per se* without overexpression of ADK is insufficient to trigger SRS (Li et al., 2008) are consistent with the lack of SRS in our ELS mice. Nevertheless, we cannot rule out the possibility that a few SE mice developed SRS since EEG recordings and western blots were performed in two distinct subsets of SE mice but not in all animals.

Using the KA model of ELS, Somera-Molina et al., 2007 reported altered hippocampal-dependent behavior and increased expression levels of Iba1 and GFAP in hippocampi of rats 1 and 40 days after KA-induced SE, respectively. This different temporal activation of the two glial populations suggests that astrogliosis may be triggered by inflammatory mediators released from microglia activation. Similarly to this report, we also found behavior deficits (impaired reference memory and sociability) and increased GFAP expression in brains of Panx1^f/f^ ELS mice 40 days after KA, suggesting that sustained astrocyte activation following ELS may underlie behavioral deficits. In support of the involvement of astrogliosis in behavioral deficits, at least in terms of social behavior, are our results showing protection against social behavior impairment in ELS NFH-Cre:Panx1^f/f^ mice which did not show alteration in GFAP expression levels. Whether the correlation between GFAP expression and sociability is or not a causal relationship should be further investigated. Nevertheless, given that there are some clinical reports indicating that the expression levels of microglia (TREM2, Cx3CR1) and astroglia markers (mainly GFAP) may contribute to autism pathology (Edmonson et al., 2014), suggest that astrocyte dysfunction may underlie the ASD phenotype observed here. In addition, given the involvement of purine nucleosides and nucleotides in astrogliosis (Hindley et al., 1994), our results suggest that neuronal Panx1 may be a major contributor that leads to astrocyte activation under these circumstances. Yet, because global deletion of Panx1 did not prevent over-expression of GFAP, we cannot exclude the possibility that mechanisms other than astrogliosis contribute to ELS-induced social behavior deficits.

In conclusion, we show here that early-life seizure induced by a single dose of KA leads to memory and social behavior deficits later in life and that deficits in sociability but not in reference memory depend on the expression of Panx1. In addition, we show that neuronal Panx1 contributes to astrogliosis and to social behavior deficits. Thus, Panx1 may be a potential therapeutic target to prevent ELS induced ASD-like behaviors.

## Supplementary Material

Supplemental Material
