# Supplementary material for: Pannexin1 Mediates Early-Life Seizure-Induced Social Behavior Deficits
Source: ASN Neuro. 2024 Jul 16;16(1):2371164. doi: 10.1080/17590914.2024.2371164 (PMC11262470; doi:10.1080/17590914.2024.2371164)
Supplement: Supplemental Material [file TASN_A_2371164_SM4338.docx]

**SUPPLEMENTARY FILES**


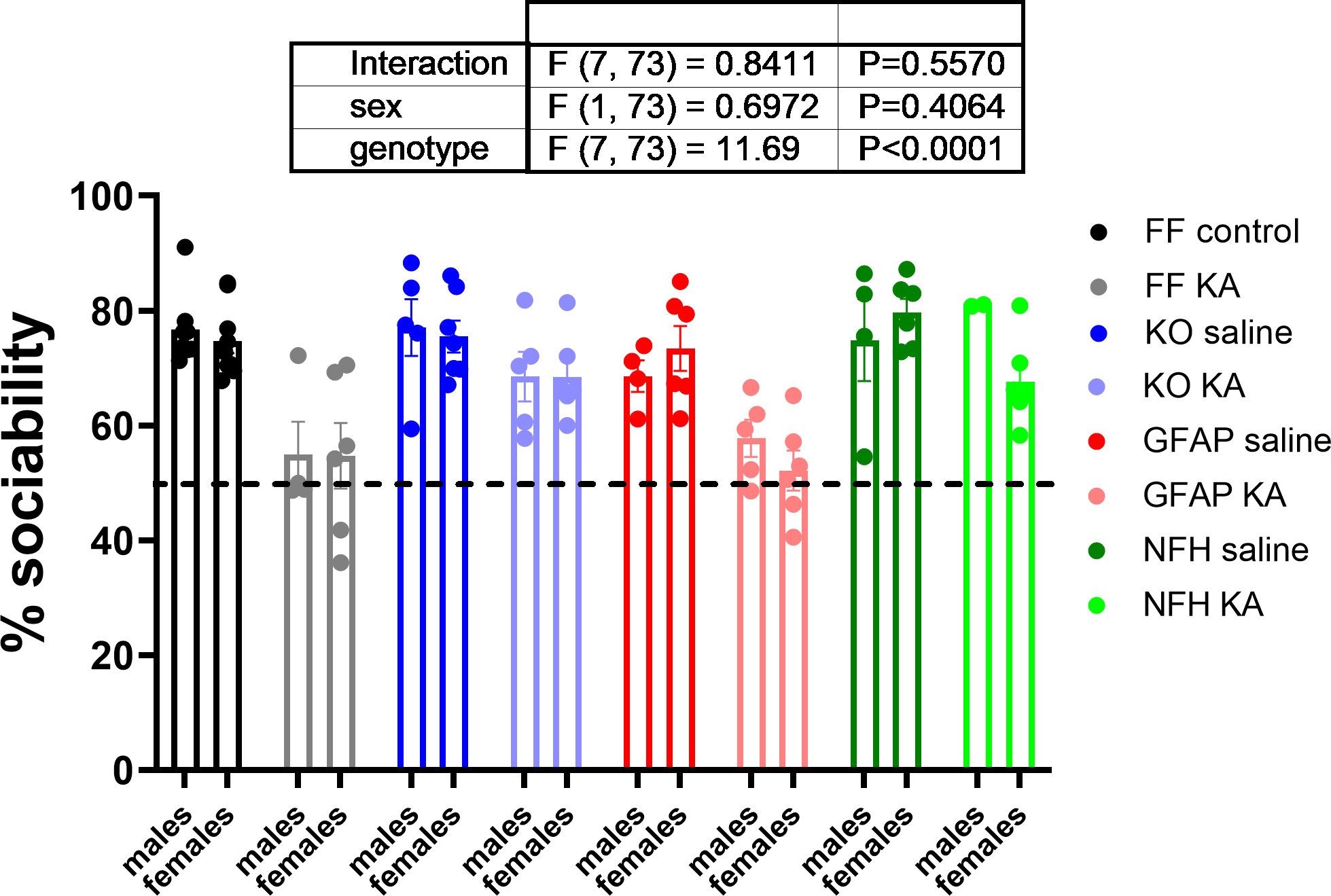


**Supplemental Figure 1S**. Sociability (percent time spent with conspecific to the total time spent with object and conspecific) obtained from two-month-old male and female mice that were injected with saline (sal) and kainic acid (KA) at P21. No significant sex differences were detected by two-way ANOVA, while significant differences in sociability were recorded between genotypes.


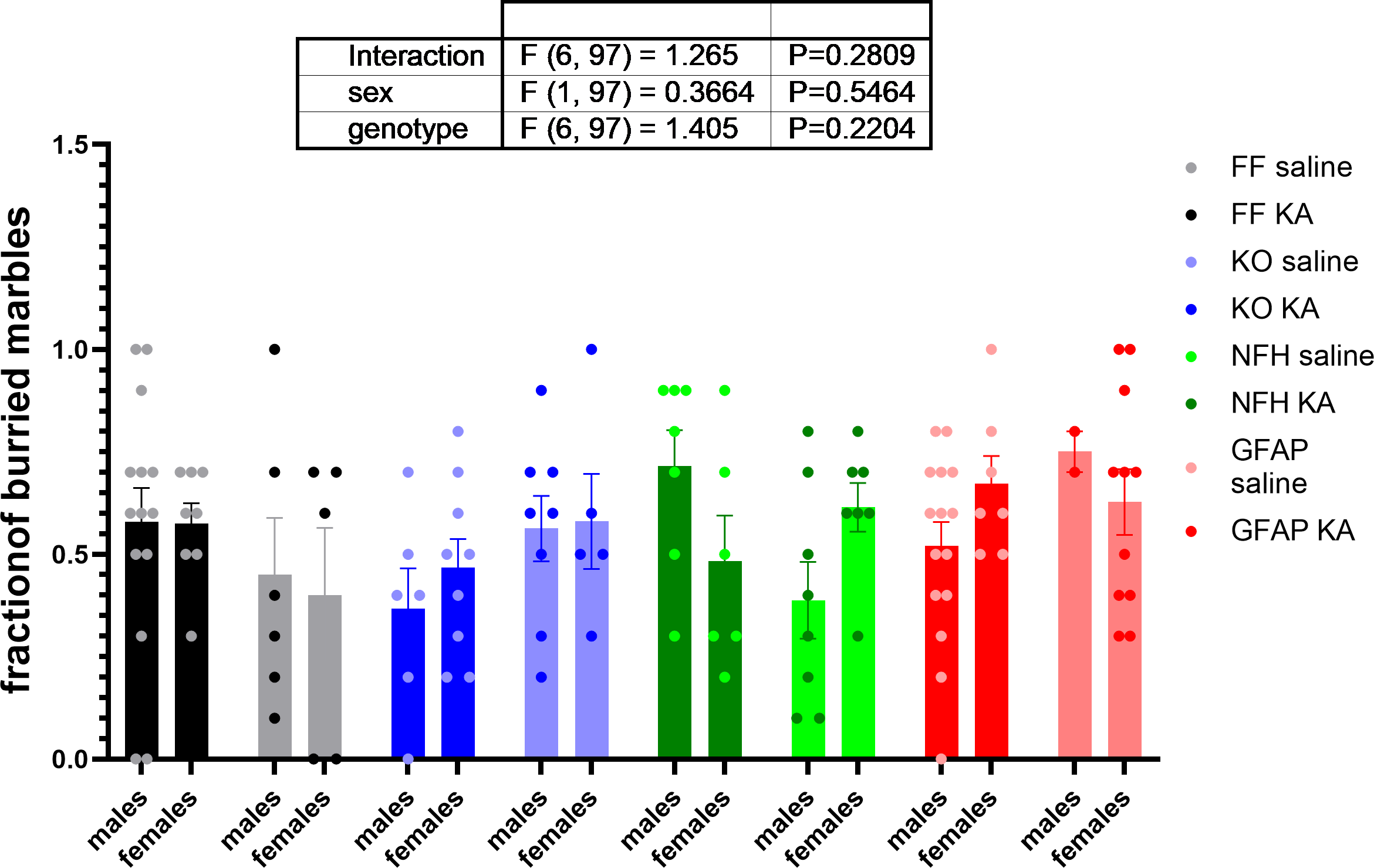


**Supplemental Figure 2S**. Fraction of buried marbles measured from 2 months old Panx1^f/f^ (FF), global Panx1 knockout (KO), NFH-Cre:Panx1^f/f^ (NFH), and GFAP-Cre:Panx1^f/f^ (GFAP) mice, injected with saline (dark colored bars) and kainic acid (light colored bars) at age P21. No significant genotype and sex differences were detected by two-way ANOVA.


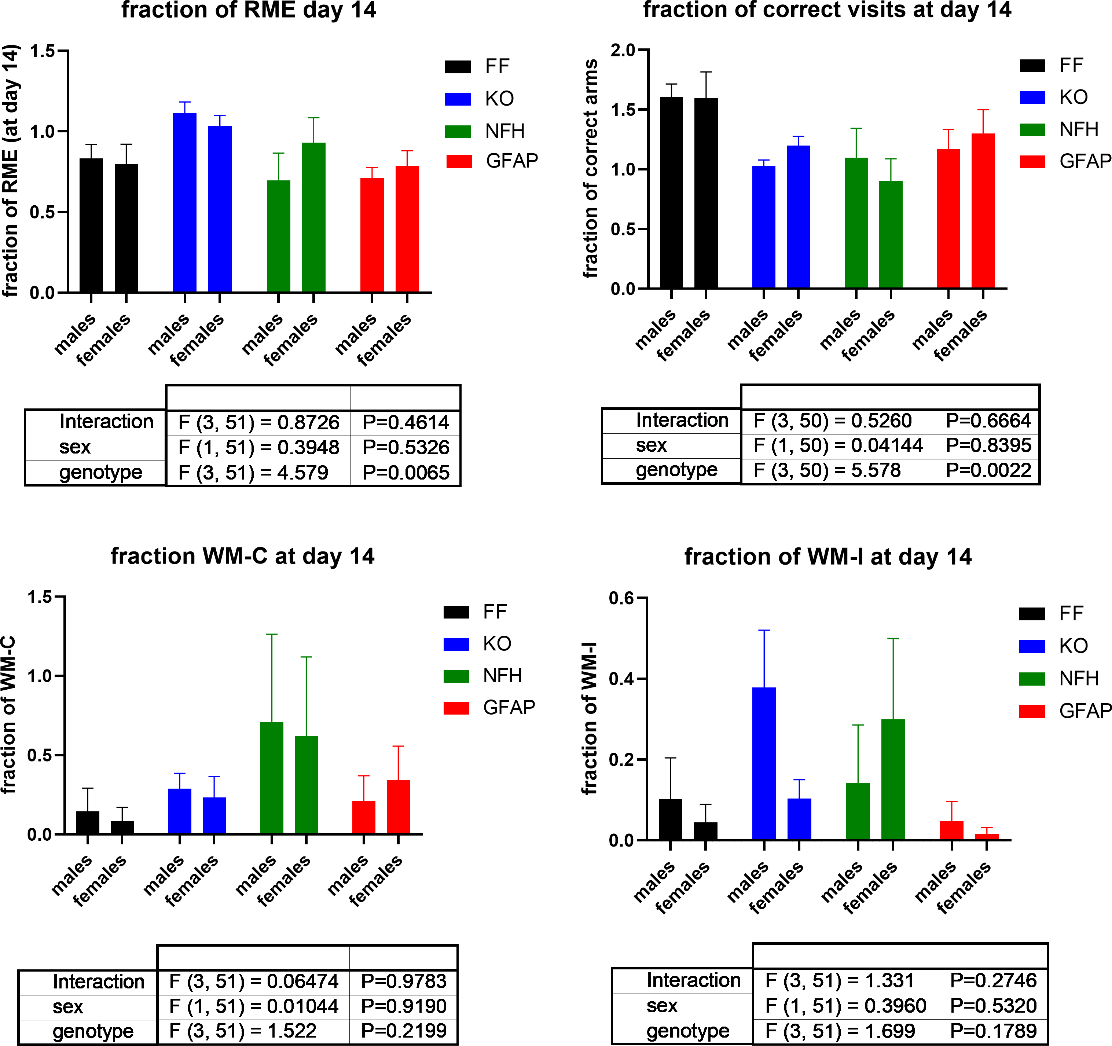


**Supplemental Figure 3S**. Fraction of (**A**) reference memory errors (RME), (**B**) correct arms, (**C**) working memory correct errors (WM-C), and of (**D**) working memory incorrect errors (WM-I) recorded from male and female Panx1^f/f^ (FF; 7 males and 8 females), global Panx1 knockout (KO; 11 males and 10 females), NFH-Cre:Panx1^f/f^ (NFH; 7 males and 5 females), and GFAP-Cre:Panx1^f/f^ (GFAP; 6 males and 5 females) mice during the test day of the 8-arms radial maze. No significant sex differences were detected by two-way ANOVA, while significant differences in RME and correct arms were recorded between genotypes.


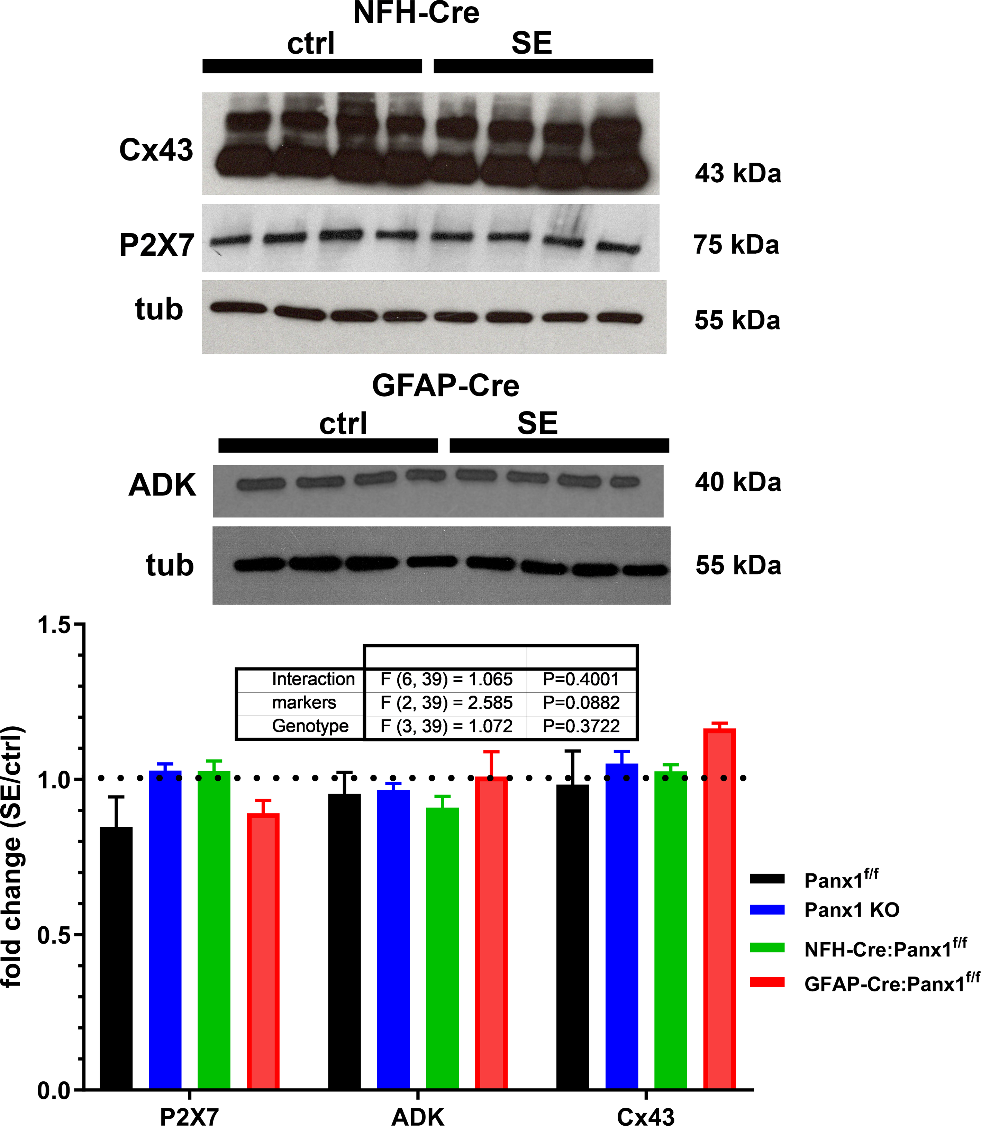


**Supplemental Figure 4S**. Western blot analysis of changes in the expression levels of P2X7, ADK and Cx43 in whole brains of 2 months old transgenic mice that were injected with KA at P21 compared to saline injected (control) ones. Two-way ANOVA indicated that there was no significant interaction between markers and genotype (p=0.40) and no significant differences among the markers (p=0.088) or genotypes (p=0.37). Examples of western blots showing the expression levels of Cx43, P2X7 and ADK in 4 saline- and 4 KA-injected NHF-Cre:Panx1^f/f^ and GFAP-Cre-Panx1^f/f^ mice are displayed on the top panel.


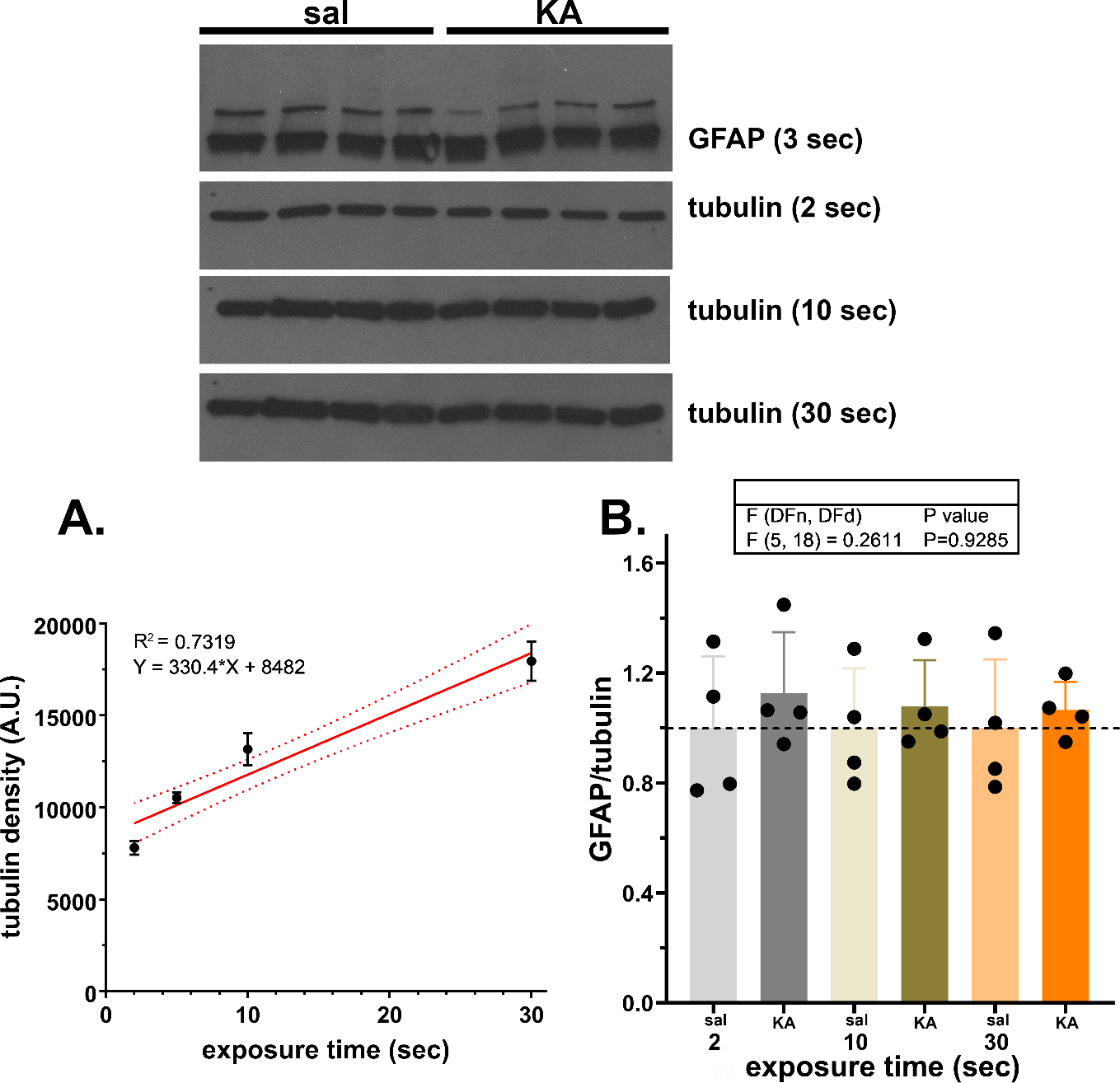


**Supplemental Figure 5S**. Effect of tubulin X-ray film exposure time on the normalized values of GFAP expression levels obtained from brain samples of NFH-Cre:Panx1^f/f^ mice injected with saline (sal) or kainic acid (KA). *Top*: Western blot images showing GFAP bands obtained from X-ray film exposed for 3 sec and of tubulin bands obtained from 3 different X-ray films exposure times (2, 10, and 30 sec). *(A)* Linear regression and 95% confidence intervals of densitometry measures obtained for tubulin bands when X-ray films were exposed for 2, 5, 10, and 30 seconds. *(B)* Relative GFAP expression levels obtained from saline (sal)- and KA-injected mice normalized to that of tubulin obtained at different X-ray film exposure times. Note that although the standard deviations of GFAP expression varied when normalized to that of tubulin obtained from different X-ray films exposure times, there is no significant difference in GFAP/tubulin levels among the groups (p = 0.9285, One Way ANOVA).
